# Sex Chromosome Effects on Male–Female Differences in Mammals

**DOI:** 10.1016/j.cub.2018.09.018

**Published:** 2018-11-19

**Authors:** Daniel M. Snell, James M.A. Turner

**Affiliations:** Sex Chromosome Biology Laboratory, The Francis Crick Institute, 1 Midland Road, London NW1 1AT, UK

## Abstract

Fundamental differences exist between males and females, encompassing anatomy, physiology, behaviour, and genetics. Such differences undoubtedly play a part in the well documented, yet poorly understood, disparity in disease susceptibility between the sexes. Although traditionally attributed to gonadal sex hormone effects, recent work has begun to shed more light on the contribution of genetics — and in particular the sex chromosomes — to these sexual dimorphisms. Here, we explore the accumulating evidence for a significant genetic component to mammalian sexual dimorphism through the paradigm of sex chromosome evolution. The differences between the extant X and Y chromosomes, at both a sequence and regulatory level, arose across 166 million years. A functional result of these differences is cell autonomous sexual dimorphism. By understanding the process that changed a pair of homologous ancestral autosomes into the extant mammalian X and Y, we believe it easier to consider the mechanisms that may contribute to hormone-independent male–female differences. We highlight key roles for genes with homologues present on both sex chromosomes, where the X-linked copy escapes X chromosome inactivation. Finally, we summarise current experimental paradigms and suggest areas for developments to further increase our understanding of cell autonomous sexual dimorphism in the context of health and disease.

## Teaser

In this review, Snell and Turner provide an overview of the genetic basis of sexual dimorphisms in mammals and discuss models to disentangle the effects of hormones versus genes in differences between the sexes.

## Main Text

### Introduction

Men and women differ in their physical appearance, indicative of an anatomical and physiological sexual dimorphism that is widespread in the natural world [1]. In primates, for example, males of *Gorilla* and *Mandrillus* taxa species are significantly larger than females; in contrast, females are generally larger than males in the *Lorisid* and *Cheirogalid* taxa [2]. Ultimately such differences must be attributed to male–female variation at the genetic level, which in turn drives the development of the gonads, and production of gonadal sex hormones *in utero*. Prior to this point of physiological differentiation, though, the sex chromosomes have already induced sex-specific aspects of organ development in the absence of gonadal sex hormones 3, 4. Subsequently, however, as mammals and their gonads do not each exist in isolation, these two variables must be separated in order to further understand their relative contributions to sexual dimorphism in human health and disease. In this review, we seek first to highlight some of the key evidence of human sexual dimorphism. Subsequently, we use the evolution of the sex chromosomes as a paradigm with which to understand the possible sources of genetic sexual dimorphism in mammals. Finally, we summarise a small number of the model systems available for investigating the mechanisms of mammalian genetic sexual dimorphism.

### Evidence for Sexual Dimorphism from Human Health and Disease

The difference in disease prevalence rates between males and females has been recognised for many years, with examples from cradle to grave. Boys are more likely to be born with pyloric stenosis or malformations of the genitourinary tract, whereas girls are more likely to have developmental dysplasia of the hip or scoliosis 5, 6. In early childhood, boys have a higher incidence of bacterial and viral infections, including meningitis, septicaemia, influenza A and respiratory syncytial viruses 7, 8, 9, 10. In adult life, autoimmune disease, depression and dementia are more common in females, whereas cardiovascular disease, schizophrenia, and Parkinson’s disease are more prevalent in males 11, 12, 13, 14, 15, 16. Although these associations have been shown to be reproducible, the underlying mechanisms are yet to be definitively elucidated. The most prominent two hypotheses attribute these sexual dimorphisms to either the gonadal sex hormones or the sex chromosomes.

### Gonadal Sex Hormones

Both male and female human fetuses are exposed to high levels of maternal oestrogens *in utero*, in addition to hormones produced by the placenta [17]. Furthermore, males start producing testosterone following testis determination at around eight weeks gestation [18]. At birth, sex hormone levels drop significantly in both sexes as the feto-placental unit is separated, before rising again transiently at around two to three months during ‘mini-puberty’ [19]. Subsequently, girls enter puberty slightly earlier than boys, and both sexes achieve maximum sex hormone levels during their mid-teens. In later life, from around the age of 50, testosterone levels in men drop gradually, whereas oestrogen levels in women fall precipitously during menopause [19].

A number of diseases have been associated with these sex-specific patterns of hormone secretion. For example, boys have a high prevalence of asthma in the pre-pubertal years [20]. Following puberty, when testosterone production is markedly increased, the burden of disease is significantly reduced. In contrast, the prevalence of asthma during childhood in girls is low, but this increases significantly during puberty, as does the risk of severe asthma [21]. Interestingly, there is a subsequent drop in asthma severity in women aged 50–65, correlating with the timing of menopause and reduced oestrogen production [21]. Post-menopausal women are also at increased risk of developing cardiovascular disease, which has similarly been attributed to reduced oestrogen levels [22]. The endocrine system therefore appears to play a significant role in mediating some sexual dimorphisms in disease. However, with our ever-increasing understanding of sex chromosomes, it has become clear that some of the differences between males and females are due to genetics.

### Genetic Causes of Sexual Dimorphism: Understanding from Evolution

Genetic testis determination triggered the evolution of the mammalian sex chromosomes, producing a pair of chromosomes fundamentally different from the autosomes in terms of gene content, regulation of gene expression, and inheritance. The extant X and Y chromosomes, and the females and males in which they exist, also differ from each other as a result of this process. We can therefore use the evolution of the sex chromosomes as a paradigm for understanding possible genetic mechanisms underlying male–female differences.

**Figure 1:**
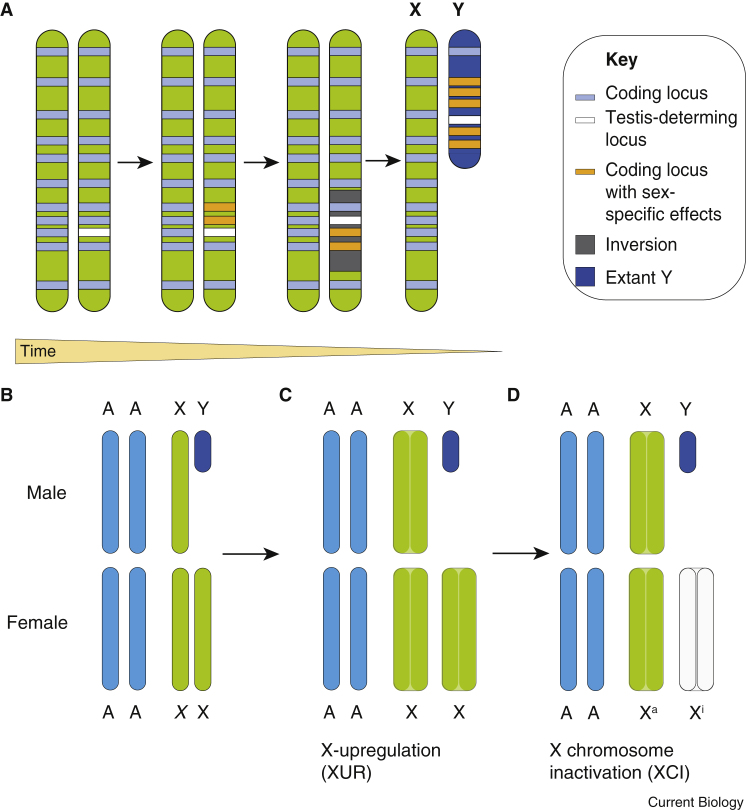
The evolution of the mammalian sex chromosomes and dosage compensation mechanisms. (A) A testis-determining locus (proto-*SRY*, white) was acquired on an autosome around 148–166 million years ago. Sexually antagonistic alleles (orange) then evolved at nearby loci, selected for in males due to their tight linkage to *SRY*. Recombination suppression between the proto-X and -Y chromosomes likely followed on from chromosomal inversions (grey), which were subsequently only carried by males. Over evolutionary time, the lack of sexual recombination led to the appearance of repetitive DNA sequences and short-term expansion. In the longer term, large deletions took place. The outcome of this process is the small, relatively gene poor Y chromosome observed in most eutherian mammals today. Concurrent with this process, X upregulation (XUR) evolved to balance X gene dosage between the single X chromosome and the autosomes in males: this is depicted as the doubled surface area of the X chromosomes in (C) compared to (B). However, XUR was passed on to XX offspring, resulting in X:autosome dosage disparity between males and females. X chromosome inactivation (XCI) then evolved to repress one of the two X chromosomes in XX cells (D). This is depicted as the loss of colour of the X chromosome. Abbreviations: Xa, active X chromosome; Xi, inactive X chromosome.

The mammalian sex chromosomes have evolved from a pair of autosomes during the past 166 million years (Figure 1A) 23, 24. Between 148 and 166 million years ago, mutations on the proto-Y chromosome resulted in the creation of the testis-determining gene *SRY*: carriers of *SRY* develop with testes, while non-carriers develop with ovaries 25, 26. *SRY*-based genetic testis determination is conserved in most eutherian mammals, and the sequence is present in metatherians [27], though whether it retains a role in testis determination in this mammalian clade remains an open question.

After the acquisition of *SRY*, the proto-Y chromosome picked up a number of male-beneficial mutations. As a result of linkage with the testis-determining locus, these mutations provided the selective force to suppress recombination between proto-X and proto-Y [28]. Mechanistically, the suppression and eventual elimination of recombination was possibly achieved by a series of local inversions [29]. Non-recombining regions also accumulated deleterious mutations that could not be repaired. Over evolutionary time, lack of recombination led to the accrual of repetitive DNA sequences and a short-term increase in the size of the chromosome, though this eventually resulted in large deletions and explains the relatively diminutive size of the Y chromosome in many mammals 28, 30. Most genes from the ancestral autosome pair were therefore lost from the Y chromosome, whereas the X chromosome largely maintained its gene content 23, 27, 31. Taking humans as an example, the extant Y chromosome encodes fewer than 78 proteins; in contrast, the X chromosome contains around 800 genes [28].

As a result of the evolution of XY testis determination, female mammals carry two copies of the relatively gene-rich X chromosome, whereas male mammals carry a single copy of the X chromosome and a gene-poor Y chromosome. The difference in X-linked gene dosage between males and females led to the appearance of compensation mechanisms aiming, firstly, to balance X expression with that of the autosomes and, secondly, to balance X expression between the homogametic (XX) and heterogametic (XY) sexes. Susumu Ohno hypothesised that X:autosome balance was achieved in males by X chromosome upregulation (XUR), the two-fold transcriptional upregulation of the X chromosome (Figure 1B,C). However, XUR alone would leave females expressing X genes at twice the level of autosomal genes. In order to correct this X:autosome imbalance, a further step is the inactivation of one X chromosome in the homogametic sex (two of the same sex chromosomes) — X chromosome inactivation (XCI, Figure 1D). In female human embryos, XCI is random, resulting in the silencing of either the maternally derived (Xm) or paternally derived X chromosome (Xp) in each cell 32, 33. The process of XCI is effected by the long non-coding RNAs *XIST* in eutherians 34, 35, 36 and *RSX* in metatherians [37]. *XIST* RNA is expressed from and coats the future inactive X chromosome. Subsequently, a number of other mechanisms lock-in the inactive state, including the histone modification H3K27 tri-methylation 38, 39, DNA methylation 40, 41, 42, and a shift in replication timing relative to the rest of the nucleus 43, 44.

The X chromosome has the potential to cause differences between males and females in a number of ways. Firstly, XCI can be skewed, resulting in preferential expression of either Xm or Xp. Secondly, a number of genes escape XCI and are thus expressed from both X chromosomes. These genes are therefore more highly expressed in XX females compared to XY males, resulting in further potential for cell autonomous sexual dimorphism. Thirdly, the parental origin of the X chromosome in males and females is not equivalent, and differential gene expression between the sexes could result from genomic imprinting.

### X Chromosome Inactivation: Mosaicism and Skewing

**Figure 2:**
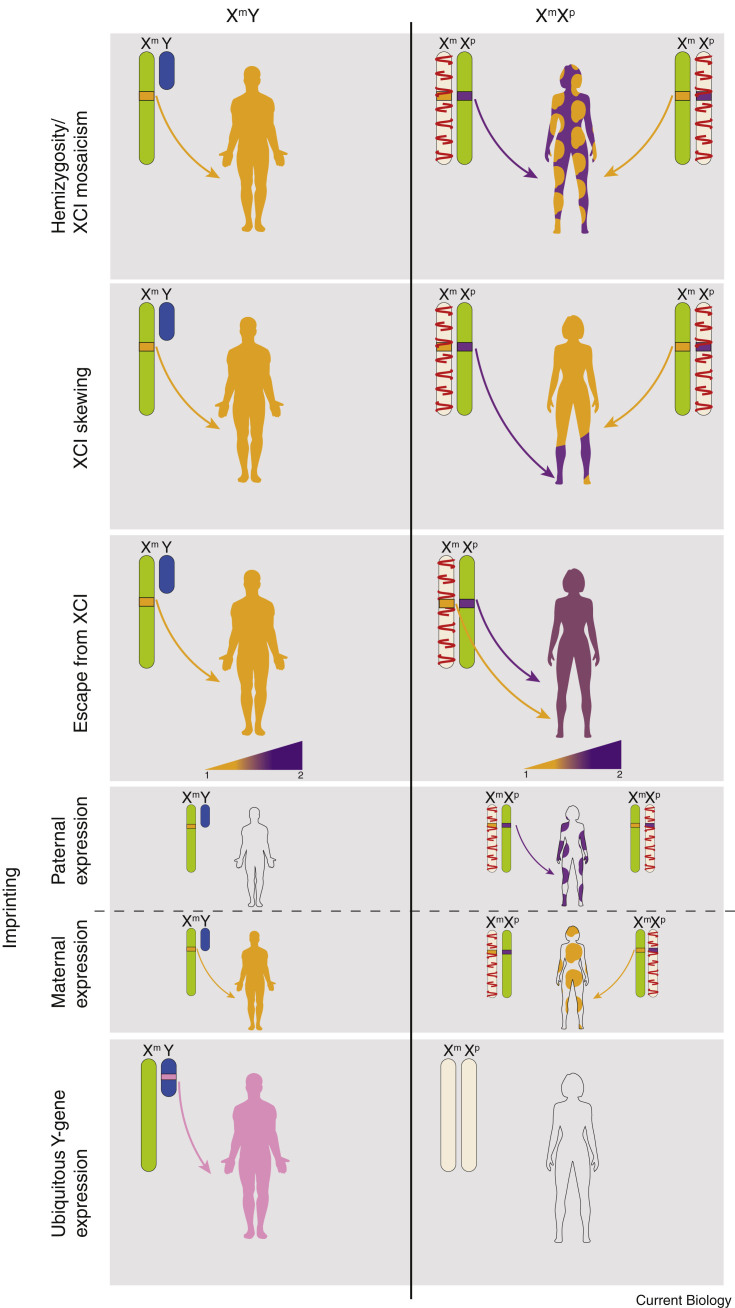
Possible mechanisms underlying male–female genetic sexual dimorphism in eutherian mammals. The organism-wide expression of an individual gene allele is represented by block colour, with XY males in the left-hand column and XX females in the right-hand column. (A) A single allele of an X-linked gene is expressed in all cells in the male, whereas due to X chromosome inactivation (XCI), the same allele is only expressed in 50% of cells in the female. (B) XCI skewing can result in a change to the percentage of cells expressing any given X allele in females. (C) As both alleles of XCI escapee genes are expressed in females, the relative expression is increased compared to males. (D) Imprinting resulting in Xp allelic expression would be absent in males due to the absence of Xp, and would be present in 50% of cells in females. Imprinting resulting in Xm allelic expression would be present in all cells in males and 50% of cells in females. (E) Ubiquitously expressed Y-linked genes are only present in males. Abbreviations: Xm, maternally derived X chromosome; Xp, paternally derived X chromosome. Gene expression is depicted in arbitrary units, taking 1 as normal expression for a single chromosome.

As a result of XCI, XX females are mosaic, with each cell expressing either Xm- or Xp-genes. A well-known representation of this phenomenon is the tortoiseshell cat, which is a mosaic of black and orange X-linked coat colours [45]. X chromosome mosaicism has long been recognised as a way in which individuals with two X chromosomes differ from those with a single X chromosome, both in terms of normal physiology and disease [46]. Physiologically, XX females express paternal X alleles in 50% of cells, whereas XY males express maternal X alleles in 100% of cells. Any subtle difference in function between the two alleles could therefore manifest as sexual dimorphism (Figure 2). Significant differences in function present as X-linked disease. In males, the presence of a single X chromosome means that X-linked recessive mutations have a fully-penetrant phenotype, but in females this is usually mild or not clinically apparent. X-linked diseases present a range of phenotypes, from relatively benign colour blindness [47], through life-limiting Duchenne and Becker muscular dystrophies [48], to embryonic lethality, as in incontinentia pigmenti [49].

Occasionally females also have a typically male disease phenotype, denoted as manifesting heterozygosity. In these individuals, XCI is no longer random, and a skew is present. Such a skew can be classified as either primary, if it arose at the onset of XCI, or secondary if it arose later [50]. There is abundant evidence for the existence of primary skewing in mouse, resulting from the influence of a locus denoted the X controlling element (Xce). Cattanach observed that certain mouse strains have stronger Xces, such that the X chromosomes carrying these Xces are more likely to remain active in F1 hybrid crosses [51]. In humans, there is little evidence for either the presence of an Xce or primary skewing. Some studies have suggested there may be a genetic component to XCI choice, i.e. inferring the existence of a human XCE 52, 53; however, more work is required to definitively address this question.

Secondary skewing has been observed at a population level in humans [53]. In a situation where no primary skew is present, two populations of cells will exist in an XX female: each expressing either the Xm or Xp allele of any given X-linked gene (Figure 2). Each of these alleles may differentially affect cellular growth, such that the rate of proliferation varies between the two populations, and a competition ensues [54]. The cell population with the growth advantage will outgrow the other — usually, but not always, the normal and mutant alleles, respectively 46, 55. Based on evidence in the literature, it is likely that this secondary skewed XCI is largely tissue-specific, and is common in normal, healthy individuals 53, 56. Skewed XCI has also been proposed as one of the causes underlying the female sex bias in autoimmune disease, and in this context it is known as the loss of mosaicism hypothesis [57].

### Escape from X Chromosome Inactivation

A number of X-linked genes are not silenced by XCI, and could therefore effect male–female differences in expression (Figure 2). Some of these genes are located within the pseudoautosomal region (PAR), and others have been found outside this region.

The PAR is an area of sequence homology between the X and Y chromosomes [58]. This homology enables X–Y pairing, synapsis, and recombination during meiosis 59, 60, 61. As males and females have PAR genes in equal copy number, it was expected that expression levels would be equivalent between the sexes. However, recent work indicates the existence of a male expression bias in humans [62]. This bias likely results from XCI spreading into the PAR on the inactive X (Xi) in females, but increased expression from the Y-linked genes in males could also contribute. The PAR is perhaps one of the most poorly studied genomic regions in sequenced eutherian mammals, as the assembly quality is not equivalent to the rest of the genome. Further work may build upon the recently reported male expression bias to reveal an unexpected role for the PAR in mammalian sexual dimorphism.

Outside of the human PAR, it has been estimated that around 12% of X genes show consistent escape, and a further 8% escape variably in different individuals and different tissues 62, 63, 64, whereas in mouse, the equivalent numbers are 3% and 4%, respectively 65, 66. The disparity in constitutive escape genes between human and mouse has been attributed to the arrangement of the genes on the chromosome. In mouse, escapees are situated in blocks of only one or two genes, whereas in human these blocks contain 10–15 genes [67]. Even within species, though, the process has inherent variability, creating the potential for sexually dimorphic effects. A recent study in humans showed that only 41% of XCI-escape genes are consistently expressed from both alleles across multiple tissues. For the remainder, inter-tissue variability in XCI-escape was observed. There was also significant variability in Xi gene expression between the two X chromosome haplotypes within an individual. Furthermore, escapee expression from the Xi was on average only one-third of the level of expression from the active X (Xa) [62]. Importantly, 52 of the 67 non-PAR escape genes showed female-biased expression. Taken together, these data suggest that the process of XCI-escape is tightly regulated for some genes and highly variable for others. This may reflect absolute limits on gene dosage for tightly regulated genes and flexibility of expression for those showing variability. The outcomes following XCI, i.e. silencing, variable escape and consistent escape, have the potential to give rise to male–female and female–female phenotypic variation, as evidenced by the female-biased expression of those consistent escapees. More work will be required to elucidate whether expression bias at the RNA level translates into phenotypic sexual dimorphism at the organism level.

Further evidence of the role of escape genes in sexual dimorphism has emerged from studies of human cancers. Many cancers show a sex bias, including those affecting the kidney and renal pelvis, blood, and brain 68, 69, 70, 71. Recent work has associated part of this bias with mutations in genes that escape XCI, so-called escape from X inactivation tumor suppressors (EXITS) [72]. By analysing 21 different tumor types, loss-of-function mutations in X genes *ATRX*, *CNKSR2*, *DDX3X*, *KDM5C*, *KDM6A*, and *MAGEC3* were found more commonly in males than females [72]. These genes could be tumor suppressors that are required in at least one copy to prevent oncogenesis. The single X gene copy in males is therefore more vulnerable to a mutation event than the two copies present in females [73]. A number of these X genes have Y homologues, which may or may not carry out the same function as the X copy (also see next section). As loss-of-function mutations in X genes were found more commonly in males, this would argue against functional conservation between X and Y genes. The study further investigated whether the Y homologue could also act as a tumor suppressor by looking at chromosome loss instead of mutations. It was found that tumors from female patients with an EXITS gene mutation lose the second X chromosome more commonly than males with an EXITS gene mutation lose the Y chromosome 74, 75, 76. This result suggested that EXITS genes were more effective tumor suppressors than their Y-linked homologues, as male patients received a cancer diagnosis without the loss of Y-linked genes. Moreover, it supports the hypothesis that X–Y gene pairs have functionally diverged, and thus contribute to male–female sexual dimorphism.

### X–Y Gene Pairs and the Y Chromosome

**Figure 3:**
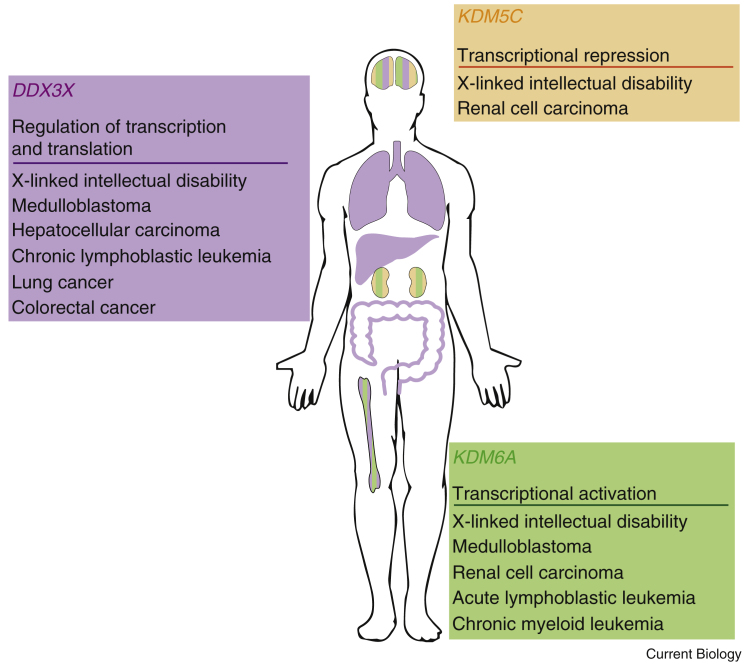
Genes escaping XCI are implicated in a range of sexually dimorphic diseases. A small number of X-linked genes show ubiquitous expression, have extant Y-linked homologues, and escape XCI. These genes have roles in the regulation of gene expression and are implicated in male–female sexual dimorphism in a wide range of diseases. Specific organs affected are indicated by the gene-related colours, i.e. *KDM5C* in orange.

Males have a Y chromosome, whereas females do not: this is the most recognisable genetic difference between the mammalian sexes and, therefore, an obvious focus for the study of male–female genetic sexual dimorphism. However, 166 million years of evolution has led the Y chromosome to become specialised for reproduction, with primary roles in testis determination and spermatogenesis [30]. These roles are reflected in gene content and expression patterns, suggesting the Y may have less of an impact on male–female differences outside of the gonad than appearances might initially suggest. For example, *SRY* drives testis determination and testis specification, resulting in gonadal sex hormone production, and a number of ampliconic genes are expressed exclusively in the testis and contribute to sperm production 23, 30. Nevertheless, a small number of single copy, ubiquitously expressed Y genes have been maintained across the mammalian group and through evolutionary time (Figure 2) 23, 27. Based on Gene Ontology annotations, these genes are involved in the regulation of transcription and translation 23, 27, functions that are acutely sensitive to haploinsufficiency [77]. Furthermore, each of these genes has an X-linked homologue that escapes XCI. It is therefore likely that this group was initially maintained on the ancestral mammalian Y chromosome in order to balance dosage between XX females and XY males 23, 27. This newly ascribed role for the Y chromosome as a ‘balancer’ could mean that the sexes are more similar, if both X and Y genes retain ancestral homologous function [78]. However, emerging evidence suggests a degree of functional divergence in such X–Y gene pairs, as mentioned previously for EXITS genes [72]. This small group of widely expressed genes is therefore of considerable interest in the investigation of male–female sexual dimorphism and the development of new therapeutics. While more work is required to more comprehensively profile the functions of the Y-linked homologues, some of the X-linked genes have already been characterised. Among these, genes *KDM5C*, *KDM6A* and *DDX3X* are of specific relevance. All three genes are conserved across almost all eutherian species and are implicated in sexually dimorphic diseases (Figure 3), including the cancers described above, and X-linked intellectual disability (XLID).

*KDM5C* is a histone lysine demethylase active at both di- and tri-methylated histone H3 position K4, and has a key role in transcriptional repression 79, 80, 81. Although widely expressed, *KDM5C* has been clinically implicated in impaired neuronal function and XLID, suggesting a key role in brain development 82, 83, 84, 85, 86, 87, 88, 89. A high proportion of reported *KDM5C* mutations primarily affect males and, when females are affected, the phenotype is generally less severe. The presence of the XLID phenotype in males suggests that the Y homologue, *KDM5D*, cannot fully compensate for loss of *KDM5C*, possibly because of divergence in expression or function of these two genes. The intermediate phenotype observed in heterozygous females carrying a wildtype copy of *KDM5C* supports the case for dosage sensitivity in this gene, implying that a single wild-type copy is unable to fully compensate for loss of the second copy.

*KDM6A* (*UTX*) is a histone lysine demethylase that catalyses removal of H3K27me2 and me3, and possibly also functions as a transcriptional activator 90, 91, 92. Similar to mutations in *KDM5C*, mutations in *KDM6A* have been reported in the context of XLID and are linked to the developmental disorder Kabuki syndrome [93]. Males with mutations in *KDM6A* show a more severe phenotype than females, supporting the hypothesis that the X–Y gene pair *KDM6A* and *KDM6D* (*UTY*) have diverged 94, 95, 96. In mice, males with mutations in *Kdm6a* survive to birth, whereas females carrying homozygous mutant alleles are lost mid-way through gestation [97]. This result suggests that *Uty* is able to partly compensate for the loss of *Kdm6a* in embryonic development. However, mutant males are born at sub-Mendelian frequency and show life-long grow deficiency relative to wild-type littermates, and heterozygous female mutants have no detectable phenotype [97]. Collectively, these data further support the case for divergence between *Kdm6a* and *Kdm6d* and, moreover, implicate this gene pair in male–female sexual dimorphism.

*DDX3X* is a member of the DEAD box protein family, with diverse roles in RNA splicing and export, translation initiation, cell cycle regulation, and apoptosis 98, 99, 100, 101, 102, 103, 104, 105, 106, 107. *DDX3X* mutations have been described in the context of XLID, almost exclusively affecting females 108, 109, though two recent cases have been described in males with predicted hypomorphic gene variants [110]. The absence of live males carrying loss-of-function mutations in *DDX3X* can be explained by an absolute requirement for the *DDX3X* protein product, which is present in heterozygous females but not in mutant males [110]. Additionally, this observation implies that *DDX3X* has functionally diverged from its Y homologue *DDX3Y*, as presence of the Y homologue does not facilitate survival. A similar picture is noted in the mouse model, whereby males carrying a mutated *Ddx3x* allele are lost earlier in embryonic development than heterozygous mutant females [111]. *DDX3X/Y* functional divergence likely contributes to phenotypic differences between males and females.

### X Imprinting

Eutherian embryos derived solely from paternal genomes (androgenotes) or from maternal genomes (gynogenotes) do not survive *in utero* development 112, 113, 114, 115. The requirement during embryogenesis for a paternal and maternal genome is explained by genomic imprinting, in which genes are expressed monoallelically in a parent-of-origin-specific manner [116]. As females inherit an X chromosome from both parents, whereas males inherit only a maternal X chromosome, imprinted expression of X-linked genes could result in phenotypic differences between the sexes regardless of XCI. For example, a gene expressed only from Xp would be expressed in half the cells in a female, but in no cells in a male. In contrast, a gene expressed only from Xm would be present in 100% of male cells but only half of female cells (Figure 2).

There is emerging evidence for X-linked imprinted genes in mammals. Any effects of X-linked imprinting may be shown more clearly in women with Turner syndrome, who have a single X chromosome (XO), than women with two X chromosomes. Women with Turner syndrome (XO) can inherit their single X chromosome paternally or maternally. Skuse and colleagues observed that XmO women had poorer social and verbal skills than XpO women [117], based on evidence from a set of neuropsychological tests. An imprinted X locus responsible for this effect has not yet been identified. These two populations also differ in their abdominal fat accumulation: XmO women show a typically male distribution of fat, whereas XpO women have a typically female fat distribution [118]. As all males carry a single Xm, whereas the Xp is inherited only by females, this result in XO females is consistent with X-linked imprinting.

In the mouse, a small number of X-linked imprinted genes have been reported, with the majority expressed in the placenta 119, 120, 121 and brain [122]. Similar to the result from humans, Davies and colleagues found that XmO female mice have poorer cognitive function than XpO female mice, as assessed by a reversal learning task. X-linked imprinted genes expressed in the placenta may underlie a growth phenotype long known to affect XpO female embryos. XpO embryos are growth retarded relative to XX littermates at preimplantation [123], egg cylinder (E7.25), and E10.5 stages of development 124, 125, 126. In contrast, XmO embryos, along with XY embryos, are developmentally advanced relative to XX littermates at E10.5 [126]. Interestingly, the ectoplacental cone (part of the early placenta) was found to have reduced volume in XpO conceptuses compared to XX controls [127]. Together, these data strongly suggest a role for parental origin of the X chromosome in mouse embryonic growth. However, despite the identification of a number of X imprinting candidates in other studies 119, 120, 121, no specific locus has yet been linked to the growth deficit phenotype.

X imprinting is still at the nascent stage of development as an area of study and, as such, accurate and appropriate models are not yet in place. Person-to-person variability clouds the picture in humans when looking at the phenotypic level, while at the molecular level, any effect size is likely to be subtle and difficult to detect. The inbred mouse model provides a greater degree of control over genetics and therefore phenotype, though with the caveat of evolutionary distance between mouse and human.

### Paradigms for Investigating Cell-Autonomous Sexual Dimorphism

In order to identify the contribution of these mechanisms to male–female differences, relative contributions from genes and gonadal sex hormones must first be teased apart. Traditionally, linkage and association analyses have been used to look for sexual dimorphism in gene expression. Initial genome-wide association studies (GWAS) neglected sex chromosome data in their analyses: the sexually dimorphic expressed quantitative trait loci (eQTLs) they identified associated with polygenic traits such as waist–hip ratio 1, 128, and bone density [129], were autosomal [130]. More recently, X-linked sexually dimorphic eQTLs have been identified, associated with height and fasting insulin [131], and genomic regulatory variation [132]. While association studies are useful for unbiased, observational work at the population level in humans, there is little room for experimental manipulation, and no control for hormonal differences. Herein lies the strength of the mouse as a model organism, which is the most genetically tractable *in vivo* system available for recapitulating areas of human health and disease.

### Mouse Models

In the study of genetic sex differences between males and females, the main confounding factor is gonadal sex hormones. An ideal experimental system would therefore facilitate the separation of these two key variables, in order to attribute phenotypic effects to either genetics or hormones. Such a system would also allow for the manipulation of sex chromosome copy number, enabling further investigation into dosage of escapees from XCI, and functional redundancy in X–Y gene pairs.

**Figure 4:**
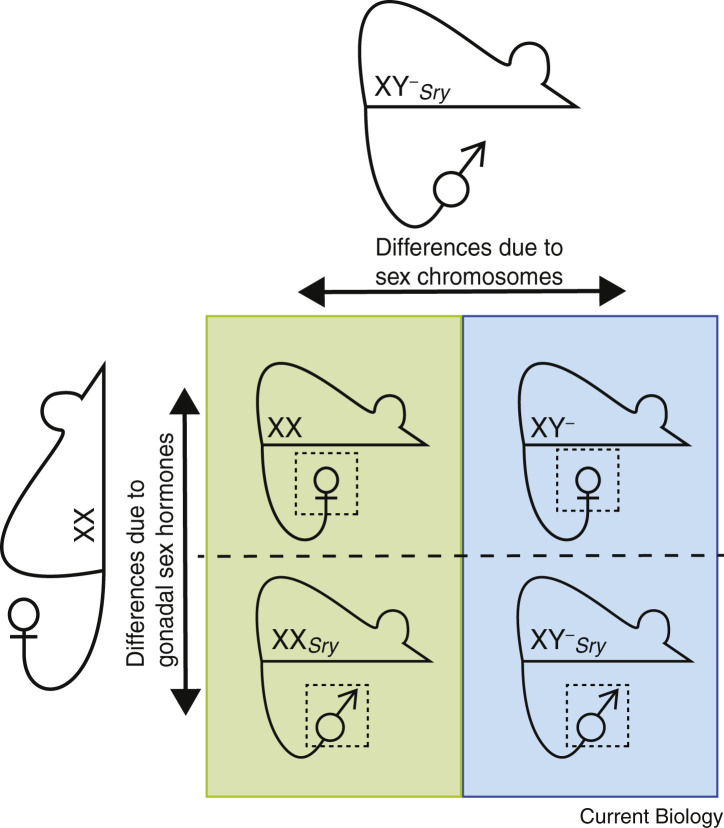
The Four Core Genotypes (FCG) model. In this Punnett square, the maternal genotype is depicted on the left-hand side and the paternal genotype is depicted at the top. A gamete from each parent carries a single sex chromosome, which come together to create the two possible offspring genotypes, XX (green column) and XY^−^ (blue column). Furthermore, the father carries *Sry* as a transgene, the inheritance of which determines the gonadal sex of the offspring: female above the dashed line (XX, XY^−^), and male below the dashed line (XX*Sry*, XY^−^*Sry*). The FCG model can therefore be used to separate sex chromosome effects (XX, green; XY, blue) from gonadal sex hormone effects (ovarian hormones above dotted line, and testicular hormones below dotted line) in mouse.

A model denoted ‘four core genotypes’ (FCG) achieves the most important aim of the ideal system. It can be used to detect XX versus XY differences that are independent of the gonad and its hormonal influence [133]. The FCG model utilises a Y chromosome with a mutated, inactive copy of *Sry* (denoted Y^−^) and an autosomal *Sry* transgene [25]. As a result, testis determination is separated from inheritance of the Y chromosome, and so XY females and XX males can be generated in addition to XX females and XY males (Figure 4).

XX and XY mice with testes have been shown to produce similar levels of gonadal sex hormones to one another, as have XX and XY mice with ovaries [134]. Phenotypic differences are therefore more likely to be attributable to sex chromosome complement 135, 136. In a further modification, the gonads can be removed (gonadectomy), which serves to reduce gonadal sex hormone levels to zero. Following this procedure, further phenotypic differences between XX and XY mice have been identified.

Despite its utility, the FCG model does not control for X chromosome copy number or the presence of a Y chromosome. For example, the XX versus XY^−^ comparison varies the number of X chromosomes, but the presence of a Y chromosome is a confounding factor. When looking to uncover the specific mechanism underlying a sex difference detected by the FCG model, a supplementary model can be used, in which a male with a variant Y chromosome (also known as XY^∗^ model) generates XX, XO, XY, and XXY offspring [137]. The effects of X chromosome copy number can be tested either in the presence of ovaries (XO versus XX) or testes (XY versus XXY), and the effects of Y chromosome presence can be tested with one (XO versus XY) or two X chromosomes (XX versus XXY). In the latter comparison, however, gonadal sex hormones are not controlled for.

Both the FCG and Y chromosome variant mouse models have been used to great effect in a number of experiments, facilitating the identification of sex differences in cardiovascular disease, metabolism and adiposity, and behaviour. For example, it is well known that men and women differ in their susceptibility to cardiovascular disease, though any mechanism has remained elusive. The FCG and Y chromosome variant models were combined with a mouse model of ischaemia reperfusion injury to mimic myocardial infarction, and removal of the gonads was performed one month prior in order to isolate sex chromosome specific effects [138]. Using both *in vivo* and *ex vivo* modelling to characterise infarct size and functional recovery, respectively, hearts from XX mice were found to perform significantly worse than hearts from XY mice, independent of gonadal sex [138]. The presence of a Y chromosome, assessed using gonadectomised variant Y chromosome model mice, seemed to have no effect on outcome. The study concluded that presence of two X chromosomes burdens the organism with greater disease risk than a single X chromosome, and therefore provides a potential focus for future human work.

The FCG model has also been used to identify sex differences in gene regulation. Euchromatic genes are silenced in a proportion of cells following translocation near to regions of heterochromatin, an effect known as position effect variegation (PEV) [139]. Using a heterochromatin-sensitive reporter transgene, Festenstein and colleagues demonstrated X chromosome dosage-dependent differences in PEV [140]. Mice with a single X chromosome showed a greater degree of silencing than those with two X chromosomes. Utilising the FCG and an XO mouse model, this effect was found to be independent of the hormonal milieu or presence of a Y chromosome. Mechanistically, it is possible that the inactive X chromosome acts as a heterochromatic sink, reducing availability of factors required for gene silencing at other heterochromatic loci [140].

Additional evidence for sexually dimorphic gene regulation has recently been observed during mammalian germ cell development. While female and male somatic cells show similar dosage compensation states in the form of XUR 141, 142, 143, the status of XUR in the germline was previously unknown. Sangrithi and colleagues (2017) found that both male and female germ cells initially exhibit upregulation of the active X chromosome. XX female germ cells then exhibited a period of X dosage excess, whereas XY male germ cells showed a period of X dosage decompensation. Interestingly, these patterns were conserved in human germ cells. In order to differentiate sex chromosome dosage effects from gonadal hormone effects, FCG and XO mouse models were used. These data revealed that like XX female germ cells, XX male germ cells had X dosage excess. Furthermore, XO female germ cells had X dosage decompensation, like XY male germ cells [144]. Mammalian germ cells therefore have an X dosage compensation state that is determined by the number of X chromosomes, and not the phenotypic sex or hormonal milieu. This contrast between X dosage compensation state and phenotypic sex could contribute to the infertility widely associated with XXY and XO genotypes and represents an interesting therapeutic angle for future work [144].

By maintaining the same autosomes and hormonal milieu, these mouse models can be used to isolate the phenotypic effects of different sex chromosome complements. However, to better understand cell autonomous differences between men and women, a human experimental system is necessary.

### Human Isogenic Cell Lines

It has been known for many years that stem cells can lose sex chromosomes when cultured *in vitro* for long periods, even when the originating clone was karyotypically normal [145]. Recent work has shown that cellular reprogramming, which generates induced pluripotent stem cells (iPSCs), also results in sex chromosome loss [146]. This process can be utilised to generate iPSC lines that are autosomally isogenic but carry different sex chromosome complements. For example, reprogramming of XXY cells generates XX, XY, XXY, and XO iPSCs. These iPSCs could potentially be screened using a multi-omics approach for differences in transcription, translation and metabolism [147] to characterise cell autonomous sexual dimorphism in the stem cell population. Additionally, iPSCs can be differentiated to study the effects of sex chromosome complement on specific cell types (i.e. as in [148]). Furthermore, mutations of interest could be introduced via genome editing to explore sexually dimorphic gene expression in a given disease model (i.e. as in [149]). Such a system would be excellent for isolating the specific effects of sex chromosome complement on gene expression at a cellular level, fully removing hormonal effects. Models of increasing complexity could then be built up, utilising multiple cell types to form tissues and introducing hormones to further understand the relationship between the two variables.

### Summary and Outlook

In 2001, the Institute of Medicine Committee on Understanding the Biology of Sex and Gender Differences reported on why sex matters in human health and disease [150], and a number of recommendations were made. Although seemingly aspirational at the time, significant progress has been made towards “determining the functions and effects of X-chromosome and Y-chromosome-linked genes in somatic cells as well as germ-line cells”. However, it was not until 2014 that the NIH mandated the consideration of sex as a biological variable in grant proposals [151], to partially address the seemingly simple recommendation “(to) determine and disclose the sex of origin of biological research materials”. In this review, we sought to build on this mandate and recommendation, providing an introduction to sex chromosome effects on male–female dimorphism, and describing a number of useful experimental models for hypothesis testing. Through fundamental sex chromosome biology, we have highlighted potential mechanisms underlying cell autonomous differences between the sexes, drawing particular attention to the role of X–Y pairs. The difference between human males and females, at the level of the DNA, is less than 80 genes on the Y chromosome. However, in order to unpick the effects of this genetic difference from that of the gonadal sex hormones and truly understand what it is to be XX versus XY, no single model provides enough data. We must use multiple models in combination. Human population work allows us to make associations between genotype and phenotype, and cell lines provide a faithful recapitulation of human physiology, but at the most basic cellular level. Animal models currently bridge the gap, allowing organism-level investigation and facilitating genetic manipulation. In order to more completely understand genetic sexual dimorphism in the context of human health and disease, we must invest in higher level models that bring together multiple cell types into tissues, tissues into organs and, eventually, organs into organisms.
